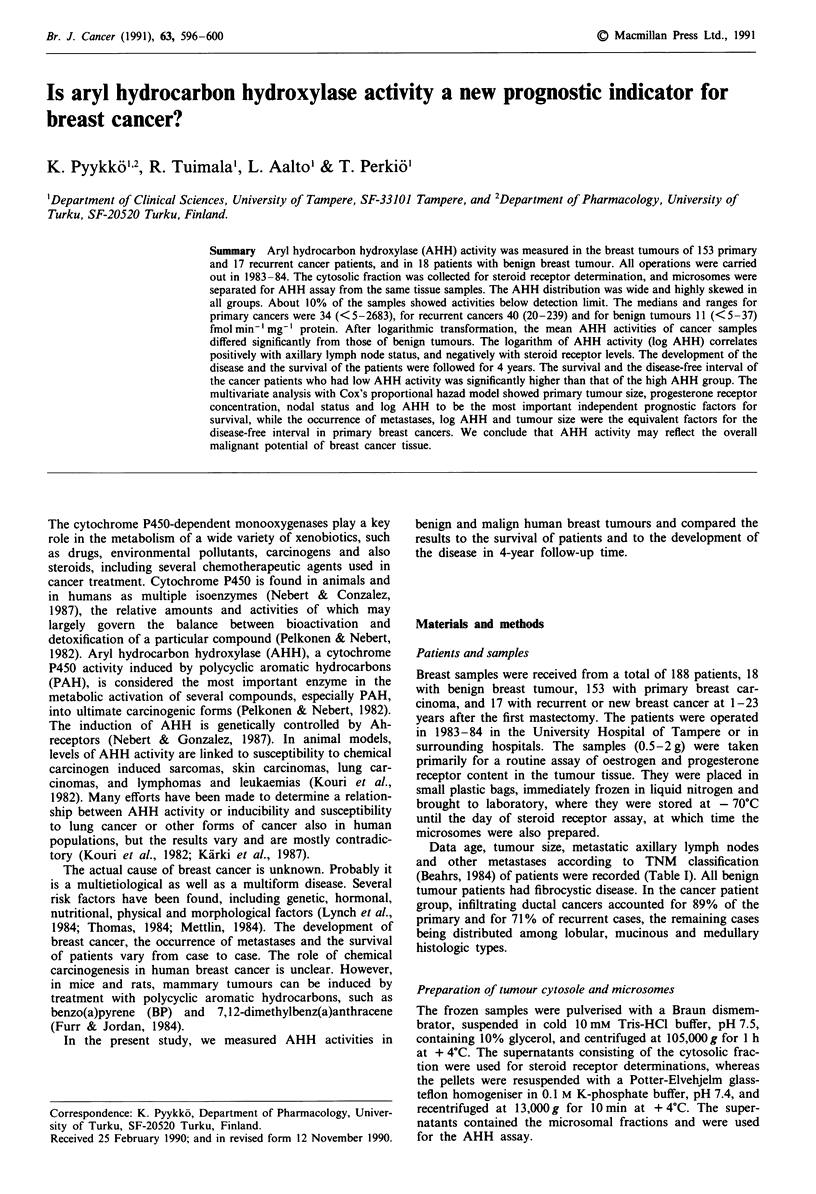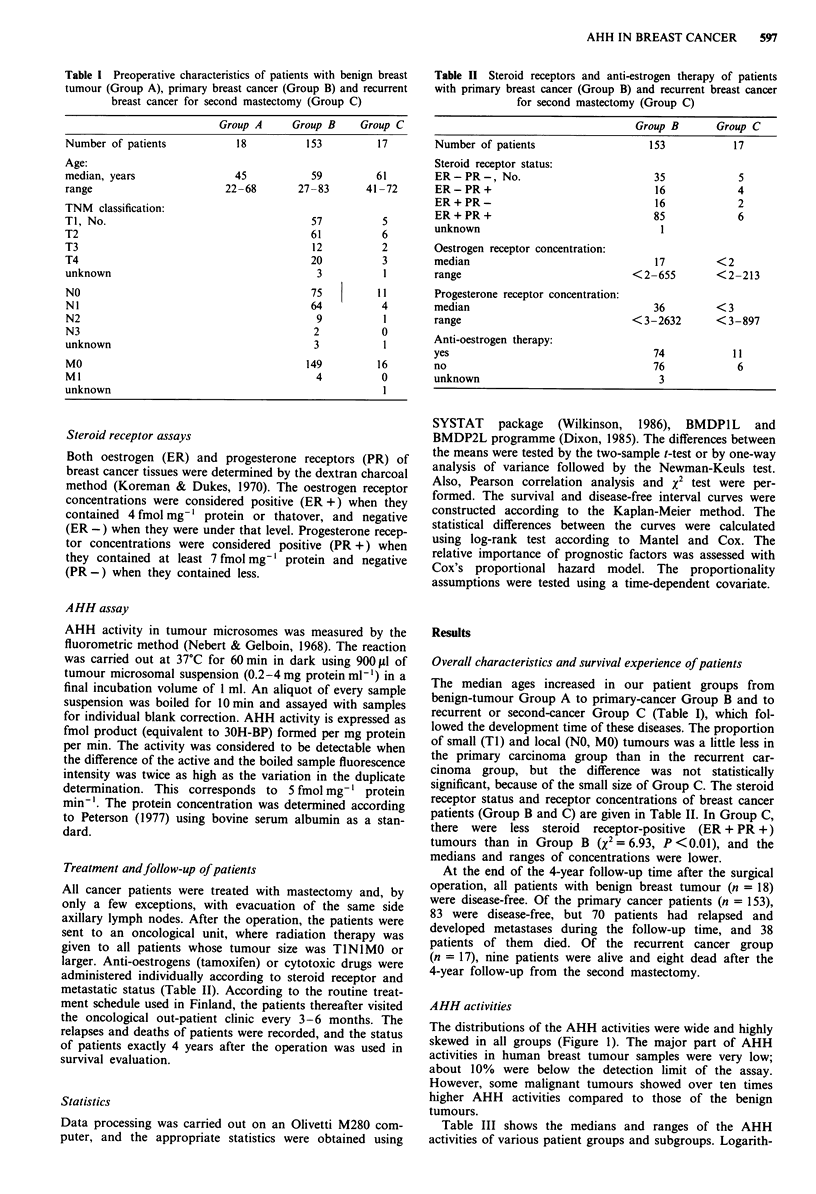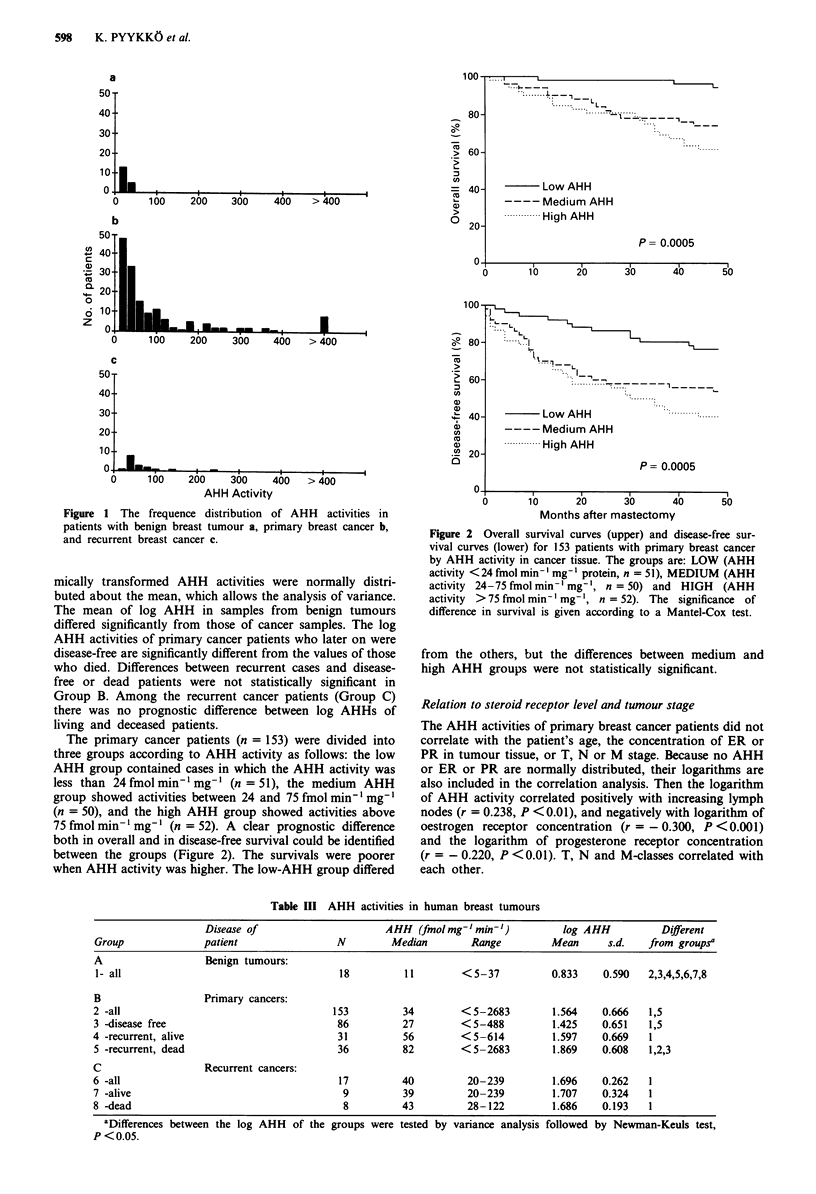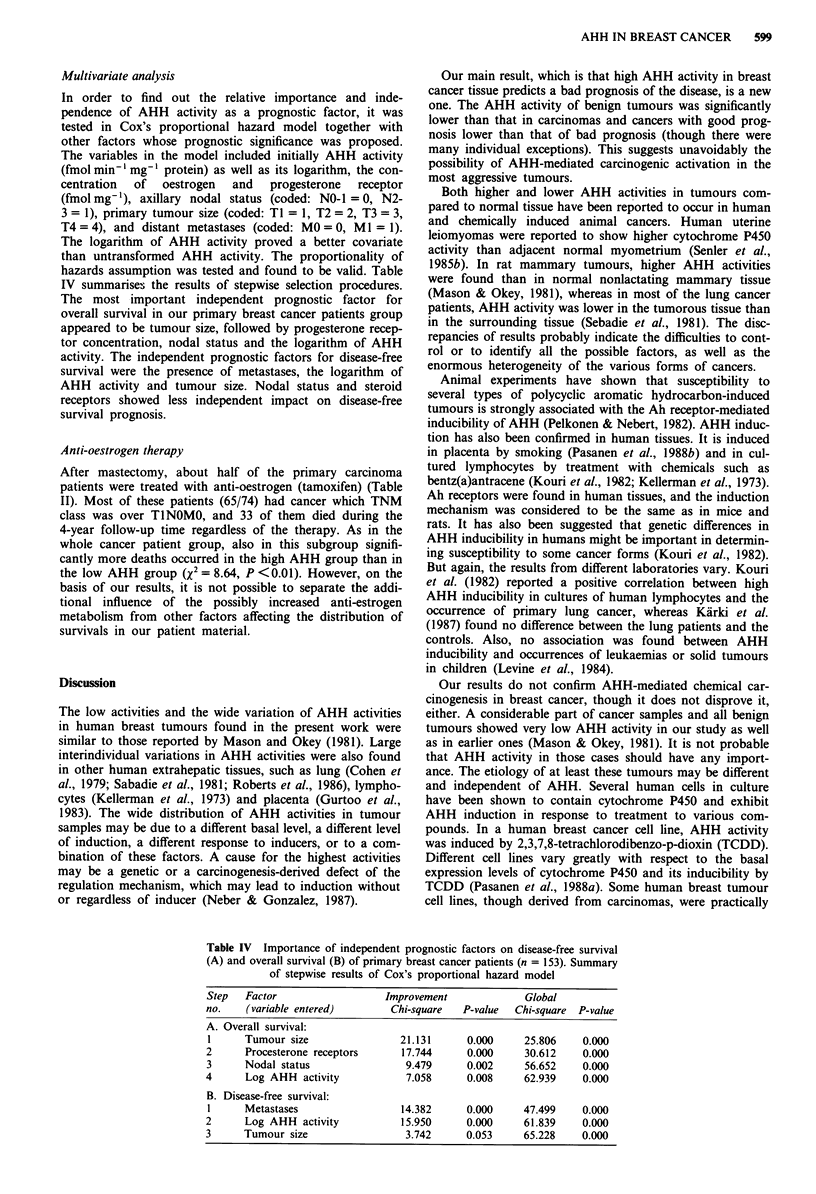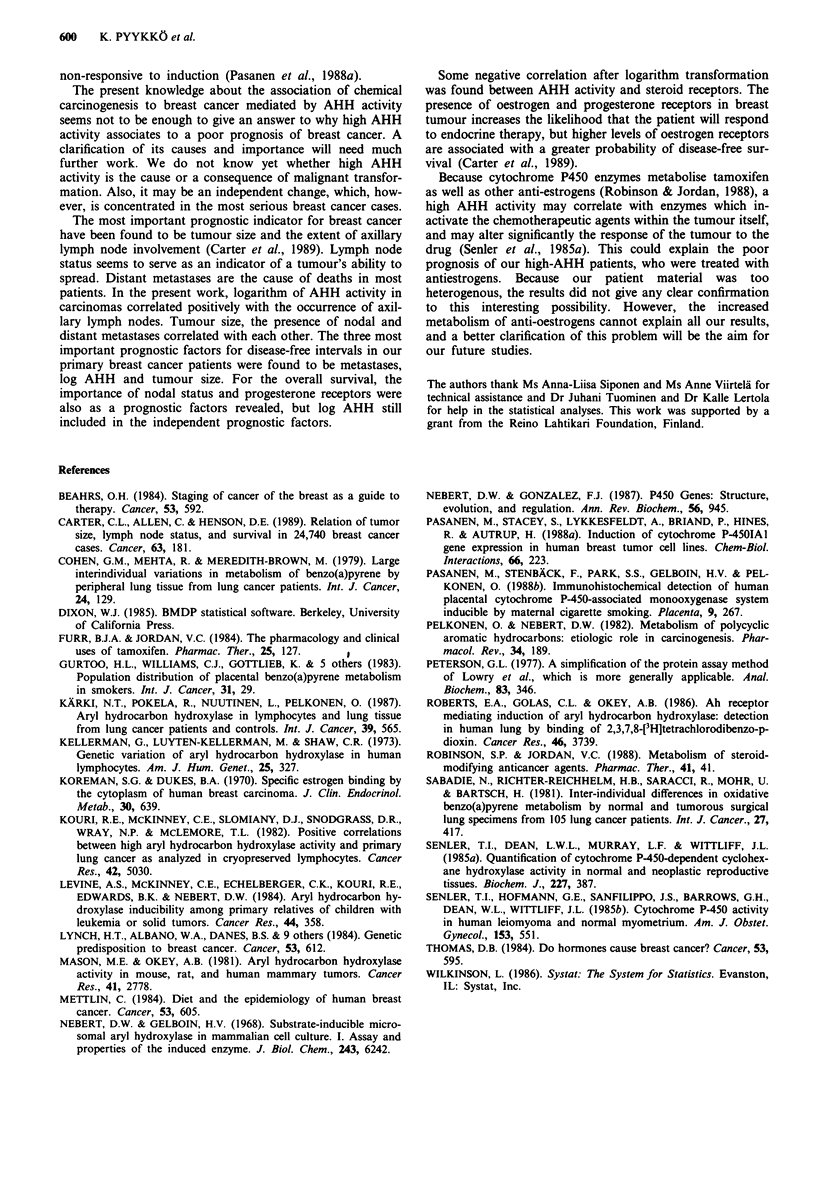# Is aryl hydrocarbon hydroxylase activity a new prognostic indicator for breast cancer?

**DOI:** 10.1038/bjc.1991.138

**Published:** 1991-04

**Authors:** K. Pyykkö, R. Tuimala, L. Aalto, T. Perkiö

**Affiliations:** Department of Clinical Sciences, University of Tampere, Finland.

## Abstract

Aryl hydrocarbon hydroxylase (AHH) activity was measured in the breast tumours of 153 primary and 17 recurrent cancer patients, and in 18 patients with benign breast tumour. All operations were carried out in 1983-84. The cytosolic fraction was collected for steroid receptor determination, and microsomes were separated for AHH assay from the same tissue samples. The AHH distribution was wide and highly skewed in all groups. About 10% of the samples showed activities below detection limit. The medians and ranges for primary cancers were 34 (less than 5-2683), for recurrent cancers 40 (20-239) and for benign tumours 11 (less than 5-37) fmol min-1 mg-1 protein. After logarithmic transformation, the mean AHH activities of cancer samples differed significantly from those of benign tumours. The logarithm of AHH activity (log AHH) correlates positively with axillary lymph node status, and negatively with steroid receptor levels. The development of the disease and the survival of the patients were followed for 4 years. The survival and the disease-free interval of the cancer patients who had low AHH activity was significantly higher than that of the high AHH group. The multivariate analysis with Cox's proportional hazad model showed primary tumour size, progesterone receptor concentration, nodal status and log AHH to be the most important independent prognostic factors for survival, while the occurrence of metastases, log AHH and tumour size were the equivalent factors for the disease-free interval in primary breast cancers. We conclude that AHH activity may reflect the overall malignant potential of breast cancer tissue.


Br. J. Cancer (1991), 63, 596-600 © Macmillan Press Ltd., 1991

### Is aryl hydrocarbon hydroxylase activity a new prognostic indicator for breast cancer?

K. Pyykkö[1,2], R. Tuimala[1], L. Aalto[1] & T. Perkiö[1]

[1]*Department of Clinical Sciences, University of Tampere, SF-33101 Tampere, and* [2]*Department of Pharmacology, University of Turku, SF-20520 Turku, Finland.*

**Summary** Aryl hydrocarbon hydroxylase (AHH) activity was measured in the breast tumours of 153 primary and 17 recurrent cancer patients, and in 18 patients with benign breast tumour. All operations were carried out in 1983-84. The cytosolic fraction was collected for steroid receptor determination, and microsomes were separated for AHH assay from the same tissue samples. The AHH distribution was wide and highly skewed in all groups. About 10% of the samples showed activities below detection limit. The medians and ranges for primary cancers were 34 (<5-2683), for recurrent cancers 40 (20-239) and for benign tumours 11 (<5-37) fmol $min^{-1}$ $mg^{-1}$ protein. After logarithmic transformation, the mean AHH activities of cancer samples differed significantly from those of benign tumours. The logarithm of AHH activity (log AHH) correlates positively with axillary lymph node status, and negatively with steroid receptor levels. The development of the disease and the survival of the patients were followed for 4 years. The survival and the disease-free interval of the cancer patients who had low AHH activity was significantly higher than that of the high AHH group. The multivariate analysis with Cox's proportional hazad model showed primary tumour size, progesterone receptor concentration, nodal status and log AHH to be the most important independent prognostic factors for survival, while the occurrence of metastases, log AHH and tumour size were the equivalent factors for the disease-free interval in primary breast cancers. We conclude that AHH activity may reflect the overall malignant potential of breast cancer tissue.

The cytochrome P450-dependent monooxygenases play a key role in the metabolism of a wide variety of xenobiotics, such as drugs, environmental pollutants, carcinogens and also steroids, including several chemotherapeutic agents used in cancer treatment. Cytochrome P450 is found in animals and in humans as multiple isoenzymes (Nebert & Conzalez, 1987), the relative amounts and activities of which may largely govern the balance between bioactivation and detoxification of a particular compound (Pelkonen & Nebert, 1982). Aryl hydrocarbon hydroxylase (AHH), a cytochrome P450 activity induced by polycyclic aromatic hydrocarbons (PAH), is considered the most important enzyme in the metabolic activation of several compounds, especially PAH, into ultimate carcinogenic forms (Pelkonen & Nebert, 1982). The induction of AHH is genetically controlled by Ah-receptors (Nebert & Gonzalez, 1987). In animal models, levels of AHH activity are linked to susceptibility to chemical carcinogen induced sarcomas, skin carcinomas, lung carcinomas, and lymphomas and leukaemias (Kouri *et al.*, 1982). Many efforts have been made to determine a relationship between AHH activity or inducibility and susceptibility to lung cancer or other forms of cancer also in human populations, but the results vary and are mostly contradictory (Kouri *et al.*, 1982; Kärki *et al.*, 1987).

The actual cause of breast cancer is unknown. Probably it is a multietiological as well as a multiform disease. Several risk factors have been found, including genetic, hormonal, nutritional, physical and morphological factors (Lynch *et al.*, 1984; Thomas, 1984; Mettlin, 1984). The development of breast cancer, the occurrence of metastases and the survival of patients vary from case to case. The role of chemical carcinogenesis in human breast cancer is unclear. However, in mice and rats, mammary tumours can be induced by treatment with polycyclic aromatic hydrocarbons, such as benzo(a)pyrene (BP) and 7,12-dimethylbenz(a)anthracene (Furr & Jordan, 1984).

In the present study, we measured AHH activities in benign and malign human breast tumours and compared the results to the survival of patients and to the development of the disease in 4-year follow-up time.

Correspondence: K. Pyykkö, Department of Pharmacology, University of Turku, SF-20520 Turku, Finland.
Received 25 February 1990; and in revised form 12 November 1990.

### Materials and methods

### *Patients and samples*

Breast samples were received from a total of 188 patients, 18 with benign breast tumour, 153 with primary breast carcinoma, and 17 with recurrent or new breast cancer at 1-23 years after the first mastectomy. The patients were operated in 1983-84 in the University Hospital of Tampere or in surrounding hospitals. The samples (0.5-2 g) were taken primarily for a routine assay of oestrogen and progesterone receptor content in the tumour tissue. They were placed in small plastic bags, immediately frozen in liquid nitrogen and brought to laboratory, where they were stored at − 70°C until the day of steroid receptor assay, at which time the microsomes were also prepared.

Data age, tumour size, metastatic axillary lymph nodes and other metastases according to TNM classification (Beahrs, 1984) of patients were recorded (Table I). All benign tumour patients had fibrocystic disease. In the cancer patient group, infiltrating ductal cancers accounted for 89% of the primary and for 71% of recurrent cases, the remaining cases being distributed among lobular, mucinous and medullary histologic types.

### *Preparation of tumour cytosole and microsomes*

The frozen samples were pulverised with a Braun dismembrator, suspended in cold 10 mM Tris-HCl buffer, pH 7.5, containing 10% glycerol, and centrifuged at 105,000 *g* for 1 h at + 4°C. The supernatants consisting of the cytosolic fraction were used for steroid receptor determinations, whereas the pellets were resuspended with a Potter-Elvehjelm glass-teflon homogeniser in 0.1 M K-phosphate buffer, pH 7.4, and recentrifuged at 13,000 *g* for 10 min at + 4°C. The supernatants contained the microsomal fractions and were used for the AHH assay.

**Table I** Preoperative characteristics of patients with benign breast tumour (Group A), primary breast cancer (Group B) and recurrent breast cancer for second mastectomy (Group C)

| | *Group A* | *Group B* | *Group C* |
|---|---|---|---|
| Number of patients | 18 | 153 | 17 |
| Age: | | | |
| median, years | 45 | 59 | 61 |
| range | 22–68 | 27–83 | 41–72 |
| TNM classification: | | | |
| T1, No. | | 57 | 5 |
| T2 | | 61 | 6 |
| T3 | | 12 | 2 |
| T4 | | 20 | 3 |
| unknown | | 3 | 1 |
| N0 | | 75 | 11 |
| N1 | | 64 | 4 |
| N2 | | 9 | 1 |
| N3 | | 2 | 0 |
| unknown | | 3 | 1 |
| M0 | | 149 | 16 |
| M1 | | 4 | 0 |
| unknown | | | 1 |

**Table II** Steroid receptors and anti-estrogen therapy of patients with primary breast cancer (Group B) and recurrent breast cancer for second mastectomy (Group C)

| | *Group B* | *Group C* |
|---|---|---|
| Number of patients | 153 | 17 |
| Steroid receptor status: | | |
| ER – PR –, No. | 35 | 5 |
| ER – PR + | 16 | 4 |
| ER + PR – | 16 | 2 |
| ER + PR + | 85 | 6 |
| unknown | 1 | |
| Oestrogen receptor concentration: | | |
| median | 17 | <2 |
| range | <2–655 | <2–213 |
| Progesterone receptor concentration: | | |
| median | 36 | <3 |
| range | <3–2632 | <3–897 |
| Anti-oestrogen therapy: | | |
| yes | 74 | 11 |
| no | 76 | 6 |
| unknown | 3 | |

*Steroid receptor assays*

Both oestrogen (ER) and progesterone receptors (PR) of breast cancer tissues were determined by the dextran charcoal method (Koreman & Dukes, 1970). The oestrogen receptor concentrations were considered positive (ER +) when they contained 4 fmol $mg^{-1}$ protein or thatover, and negative (ER –) when they were under that level. Progesterone receptor concentrations were considered positive (PR +) when they contained at least 7 fmol $mg^{-1}$ protein and negative (PR –) when they contained less.

*AHH assay*

AHH activity in tumour microsomes was measured by the fluorometric method (Nebert & Gelboin, 1968). The reaction was carried out at 37°C for 60 min in dark using 900 µl of tumour microsomal suspension (0.2–4 mg protein $ml^{-1}$) in a final incubation volume of 1 ml. An aliquot of every sample suspension was boiled for 10 min and assayed with samples for individual blank correction. AHH activity is expressed as fmol product (equivalent to 3OH-BP) formed per mg protein per min. The activity was considered to be detectable when the difference of the active and the boiled sample fluorescence intensity was twice as high as the variation in the duplicate determination. This corresponds to 5 fmol $mg^{-1}$ protein $min^{-1}$. The protein concentration was determined according to Peterson (1977) using bovine serum albumin as a standard.

*Treatment and follow-up of patients*

All cancer patients were treated with mastectomy and, by only a few exceptions, with evacuation of the same side axillary lymph nodes. After the operation, the patients were sent to an oncological unit, where radiation therapy was given to all patients whose tumour size was T1N1M0 or larger. Anti-oestrogens (tamoxifen) or cytotoxic drugs were administered individually according to steroid receptor and metastatic status (Table II). According to the routine treatment schedule used in Finland, the patients thereafter visited the oncological out-patient clinic every 3–6 months. The relapses and deaths of patients were recorded, and the status of patients exactly 4 years after the operation was used in survival evaluation.

*Statistics*

Data processing was carried out on an Olivetti M280 computer, and the appropriate statistics were obtained using SYSTAT package (Wilkinson, 1986), BMDP1L and BMDP2L programme (Dixon, 1985). The differences between the means were tested by the two-sample *t*-test or by one-way analysis of variance followed by the Newman-Keuls test. Also, Pearson correlation analysis and $\chi^2$ test were performed. The survival and disease-free interval curves were constructed according to the Kaplan-Meier method. The statistical differences between the curves were calculated using log-rank test according to Mantel and Cox. The relative importance of prognostic factors was assessed with Cox's proportional hazard model. The proportionality assumptions were tested using a time-dependent covariate.

### Results

*Overall characteristics and survival experience of patients*

The median ages increased in our patient groups from benign-tumour Group A to primary-cancer Group B and to recurrent or second-cancer Group C (Table I), which followed the development time of these diseases. The proportion of small (T1) and local (N0, M0) tumours was a little less in the primary carcinoma group than in the recurrent carcinoma group, but the difference was not statistically significant, because of the small size of Group C. The steroid receptor status and receptor concentrations of breast cancer patients (Group B and C) are given in Table II. In Group C, there were less steroid receptor-positive (ER + PR +) tumours than in Group B ($\chi^2 = 6.93$, $P < 0.01$), and the medians and ranges of concentrations were lower.

At the end of the 4-year follow-up time after the surgical operation, all patients with benign breast tumour ($n = 18$) were disease-free. Of the primary cancer patients ($n = 153$), 83 were disease-free, but 70 patients had relapsed and developed metastases during the follow-up time, and 38 patients of them died. Of the recurrent cancer group ($n = 17$), nine patients were alive and eight dead after the 4-year follow-up from the second mastectomy.

*AHH activities*

The distributions of the AHH activities were wide and highly skewed in all groups (Figure 1). The major part of AHH activities in human breast tumour samples were very low; about 10% were below the detection limit of the assay. However, some malignant tumours showed over ten times higher AHH activities compared to those of the benign tumours.

Table III shows the medians and ranges of the AHH activities of various patient groups and subgroups. Logarith-

Figure 1 The frequence distribution of AHH activities in patients with benign breast tumour a, primary breast cancer b, and recurrent breast cancer c.

Figure 2 Overall survival curves (upper) and disease-free survival curves (lower) for 153 patients with primary breast cancer by AHH activity in cancer tissue. The groups are: LOW (AHH activity <24 fmol $min^{-1}$ $mg^{-1}$ protein, $n = 51$), MEDIUM (AHH activity 24–75 fmol $min^{-1}$ $mg^{-1}$, $n = 50$) and HIGH (AHH activity >75 fmol $min^{-1}$ $mg^{-1}$, $n = 52$). The significance of difference in survival is given according to a Mantel-Cox test.

mically transformed AHH activities were normally distributed about the mean, which allows the analysis of variance. The mean of log AHH in samples from benign tumours differed significantly from those of cancer samples. The log AHH activities of primary cancer patients who later on were disease-free are significantly different from the values of those who died. Differences between recurrent cases and disease-free or dead patients were not statistically significant in Group B. Among the recurrent cancer patients (Group C) there was no prognostic difference between log AHHs of living and deceased patients.

The primary cancer patients ($n = 153$) were divided into three groups according to AHH activity as follows: the low AHH group contained cases in which the AHH activity was less than 24 fmol $min^{-1}$ $mg^{-1}$ ($n = 51$), the medium AHH group showed activities between 24 and 75 fmol $min^{-1}$ $mg^{-1}$ ($n = 50$), and the high AHH group showed activities above 75 fmol $min^{-1}$ $mg^{-1}$ ($n = 52$). A clear prognostic difference both in overall and in disease-free survival could be identified between the groups (Figure 2). The survivals were poorer when AHH activity was higher. The low-AHH group differed from the others, but the differences between medium and high AHH groups were not statistically significant.

*Relation to steroid receptor level and tumour stage*

The AHH activities of primary breast cancer patients did not correlate with the patient's age, the concentration of ER or PR in tumour tissue, or T, N or M stage. Because no AHH or ER or PR are normally distributed, their logarithms are also included in the correlation analysis. Then the logarithm of AHH activity correlated positively with increasing lymph nodes ($r = 0.238$, $P < 0.01$), and negatively with logarithm of oestrogen receptor concentration ($r = -0.300$, $P < 0.001$) and the logarithm of progesterone receptor concentration ($r = -0.220$, $P < 0.01$). T, N and M-classes correlated with each other.

**Table III** AHH activities in human breast tumours

| *Group* | *Disease of patient* | *N* | *AHH (fmol $mg^{-1}$ $min^{-1}$)* *Median* | *Range* | *log AHH* *Mean* | *s.d.* | *Different from groups*[a] |
|---|---|---|---|---|---|---|---|
| A | Benign tumours: | | | | | | |
| 1- all | | 18 | 11 | <5–37 | 0.833 | 0.590 | 2,3,4,5,6,7,8 |
| B | Primary cancers: | | | | | | |
| 2 -all | | 153 | 34 | <5–2683 | 1.564 | 0.666 | 1,5 |
| 3 -disease free | | 86 | 27 | <5–488 | 1.425 | 0.651 | 1,5 |
| 4 -recurrent, alive | | 31 | 56 | <5–614 | 1.597 | 0.669 | 1 |
| 5 -recurrent, dead | | 36 | 82 | <5–2683 | 1.869 | 0.608 | 1,2,3 |
| C | Recurrent cancers: | | | | | | |
| 6 -all | | 17 | 40 | 20–239 | 1.696 | 0.262 | 1 |
| 7 -alive | | 9 | 39 | 20–239 | 1.707 | 0.324 | 1 |
| 8 -dead | | 8 | 43 | 28–122 | 1.686 | 0.193 | 1 |

[a]Differences between the log AHH of the groups were tested by variance analysis followed by Newman-Keuls test, $P < 0.05$.

*Multivariate analysis*

In order to find out the relative importance and independence of AHH activity as a prognostic factor, it was tested in Cox's proportional hazard model together with other factors whose prognostic significance was proposed. The variables in the model included initially AHH activity (fmol $min^{-1}$ $mg^{-1}$ protein) as well as its logarithm, the concentration of oestrogen and progesterone receptor (fmol $mg^{-1}$), axillary nodal status (coded: N0-1 = 0, N2-3 = 1), primary tumour size (coded: T1 = 1, T2 = 2, T3 = 3, T4 = 4), and distant metastases (coded: M0 = 0, M1 = 1). The logarithm of AHH activity proved a better covariate than untransformed AHH activity. The proportionality of hazards assumption was tested and found to be valid. Table IV summarises the results of stepwise selection procedures. The most important independent prognostic factor for overall survival in our primary breast cancer patients group appeared to be tumour size, followed by progesterone receptor concentration, nodal status and the logarithm of AHH activity. The independent prognostic factors for disease-free survival were the presence of metastases, the logarithm of AHH activity and tumour size. Nodal status and steroid receptors showed less independent impact on disease-free survival prognosis.

*Anti-oestrogen therapy*

After mastectomy, about half of the primary carcinoma patients were treated with anti-oestrogen (tamoxifen) (Table II). Most of these patients (65/74) had cancer which TNM class was over T1N0M0, and 33 of them died during the 4-year follow-up time regardless of the therapy. As in the whole cancer patient group, also in this subgroup significantly more deaths occurred in the high AHH group than in the low AHH group ($\chi^2 = 8.64$, $P < 0.01$). However, on the basis of our results, it is not possible to separate the additional influence of the possibly increased anti-estrogen metabolism from other factors affecting the distribution of survivals in our patient material.

### Discussion

The low activities and the wide variation of AHH activities in human breast tumours found in the present work were similar to those reported by Mason and Okey (1981). Large interindividual variations in AHH activities were also found in other human extrahepatic tissues, such as lung (Cohen *et al.*, 1979; Sabadie *et al.*, 1981; Roberts *et al.*, 1986), lymphocytes (Kellerman *et al.*, 1973) and placenta (Gurtoo *et al.*, 1983). The wide distribution of AHH activities in tumour samples may be due to a different basal level, a different level of induction, a different response to inducers, or to a combination of these factors. A cause for the highest activities may be a genetic or a carcinogenesis-derived defect of the regulation mechanism, which may lead to induction without or regardless of inducer (Neber & Gonzalez, 1987).

Our main result, which is that high AHH activity in breast cancer tissue predicts a bad prognosis of the disease, is a new one. The AHH activity of benign tumours was significantly lower than that in carcinomas and cancers with good prognosis lower than that of bad prognosis (though there were many individual exceptions). This suggests unavoidably the possibility of AHH-mediated carcinogenic activation in the most aggressive tumours.

Both higher and lower AHH activities in tumours compared to normal tissue have been reported to occur in human and chemically induced animal cancers. Human uterine leiomyomas were reported to show higher cytochrome P450 activity than adjacent normal myometrium (Senler *et al.*, 1985*b*). In rat mammary tumours, higher AHH activities were found than in normal nonlactating mammary tissue (Mason & Okey, 1981), whereas in most of the lung cancer patients, AHH activity was lower in the tumorous tissue than in the surrounding tissue (Sebadie *et al.*, 1981). The discrepancies of results probably indicate the difficulties to control or to identify all the possible factors, as well as the enormous heterogeneity of the various forms of cancers.

Animal experiments have shown that susceptibility to several types of polycyclic aromatic hydrocarbon-induced tumours is strongly associated with the Ah receptor-mediated inducibility of AHH (Pelkonen & Nebert, 1982). AHH induction has also been confirmed in human tissues. It is induced in placenta by smoking (Pasanen *et al.*, 1988*b*) and in cultured lymphocytes by treatment with chemicals such as bentz(a)antracene (Kouri *et al.*, 1982; Kellerman *et al.*, 1973). Ah receptors were found in human tissues, and the induction mechanism was considered to be the same as in mice and rats. It has also been suggested that genetic differences in AHH inducibility in humans might be important in determining susceptibility to some cancer forms (Kouri *et al.*, 1982). But again, the results from different laboratories vary. Kouri *et al.* (1982) reported a positive correlation between high AHH inducibility in cultures of human lymphocytes and the occurrence of primary lung cancer, whereas Kärki *et al.* (1987) found no difference between the lung patients and the controls. Also, no association was found between AHH inducibility and occurrences of leukaemias or solid tumours in children (Levine *et al.*, 1984).

Our results do not confirm AHH-mediated chemical carcinogenesis in breast cancer, though it does not disprove it, either. A considerable part of cancer samples and all benign tumours showed very low AHH activity in our study as well as in earlier ones (Mason & Okey, 1981). It is not probable that AHH activity in those cases should have any importance. The etiology of at least these tumours may be different and independent of AHH. Several human cells in culture have been shown to contain cytochrome P450 and exhibit AHH induction in response to treatment to various compounds. In a human breast cancer cell line, AHH activity was induced by 2,3,7,8-tetrachlorodibenzo-p-dioxin (TCDD). Different cell lines vary greatly with respect to the basal expression levels of cytochrome P450 and its inducibility by TCDD (Pasanen *et al.*, 1988*a*). Some human breast tumour cell lines, though derived from carcinomas, were practically

**Table IV** Importance of independent prognostic factors on disease-free survival (A) and overall survival (B) of primary breast cancer patients ($n$ = 153). Summary of stepwise results of Cox's proportional hazard model

| *Step no.* | *Factor (variable entered)* | *Improvement Chi-square* | *P-value* | *Global Chi-square* | *P-value* |
|---|---|---|---|---|---|
| A. Overall survival: | | | | | |
| 1 | Tumour size | 21.131 | 0.000 | 25.806 | 0.000 |
| 2 | Procesterone receptors | 17.744 | 0.000 | 30.612 | 0.000 |
| 3 | Nodal status | 9.479 | 0.002 | 56.652 | 0.000 |
| 4 | Log AHH activity | 7.058 | 0.008 | 62.939 | 0.000 |
| B. Disease-free survival: | | | | | |
| 1 | Metastases | 14.382 | 0.000 | 47.499 | 0.000 |
| 2 | Log AHH activity | 15.950 | 0.000 | 61.839 | 0.000 |
| 3 | Tumour size | 3.742 | 0.053 | 65.228 | 0.000 |

non-responsive to induction (Pasanen et al., 1988a).

The present knowledge about the association of chemical carcinogenesis to breast cancer mediated by AHH activity seems not to be enough to give an answer to why high AHH activity associates to a poor prognosis of breast cancer. A clarification of its causes and importance will need much further work. We do not know yet whether high AHH activity is the cause or a consequence of malignant transformation. Also, it may be an independent change, which, however, is concentrated in the most serious breast cancer cases.

The most important prognostic indicator for breast cancer have been found to be tumour size and the extent of axillary lymph node involvement (Carter et al., 1989). Lymph node status seems to serve as an indicator of a tumour's ability to spread. Distant metastases are the cause of deaths in most patients. In the present work, logarithm of AHH activity in carcinomas correlated positively with the occurrence of axillary lymph nodes. Tumour size, the presence of nodal and distant metastases correlated with each other. The three most important prognostic factors for disease-free intervals in our primary breast cancer patients were found to be metastases, log AHH and tumour size. For the overall survival, the importance of nodal status and progesterone receptors were also as a prognostic factors revealed, but log AHH still included in the independent prognostic factors.

Some negative correlation after logarithm transformation was found between AHH activity and steroid receptors. The presence of oestrogen and progesterone receptors in breast tumour increases the likelihood that the patient will respond to endocrine therapy, but higher levels of oestrogen receptors are associated with a greater probability of disease-free survival (Carter et al., 1989).

Because cytochrome P450 enzymes metabolise tamoxifen as well as other anti-estrogens (Robinson & Jordan, 1988), a high AHH activity may correlate with enzymes which inactivate the chemotherapeutic agents within the tumour itself, and may alter significantly the response of the tumour to the drug (Senler et al., 1985a). This could explain the poor prognosis of our high-AHH patients, who were treated with antiestrogens. Because our patient material was too heterogenous, the results did not give any clear confirmation to this interesting possibility. However, the increased metabolism of anti-oestrogens cannot explain all our results, and a better clarification of this problem will be the aim for our future studies.

The authors thank Ms Anna-Liisa Siponen and Ms Anne Viirtelä for technical assistance and Dr Juhani Tuominen and Dr Kalle Lertola for help in the statistical analyses. This work was supported by a grant from the Reino Lahtikari Foundation, Finland.

### References

BEAHRS, O.H. (1984). Staging of cancer of the breast as a guide to therapy. *Cancer*, **53**, 592.

CARTER, C.L., ALLEN, C. & HENSON, D.E. (1989). Relation of tumor size, lymph node status, and survival in 24,740 breast cancer cases. *Cancer*, **63**, 181.

COHEN, G.M., MEHTA, R. & MEREDITH-BROWN, M. (1979). Large interindividual variations in metabolism of benzo(a)pyrene by peripheral lung tissue from lung cancer patients. *Int. J. Cancer*, **24**, 129.

DIXON, W.J. (1985). BMDP statistical software. Berkeley, University of California Press.

FURR, B.J.A. & JORDAN, V.C. (1984). The pharmacology and clinical uses of tamoxifen. *Pharmac. Ther.*, **25**, 127.

GURTOO, H.L., WILLIAMS, C.J., GOTTLIEB, K. & 5 others (1983). Population distribution of placental benzo(a)pyrene metabolism in smokers. *Int. J. Cancer*, **31**, 29.

KÄRKI, N.T., POKELA, R., NUUTINEN, L., PELKONEN, O. (1987). Aryl hydrocarbon hydroxylase in lymphocytes and lung tissue from lung cancer patients and controls. *Int. J. Cancer*, **39**, 565.

KELLERMAN, G., LUYTEN-KELLERMAN, M. & SHAW, C.R. (1973). Genetic variation of aryl hydrocarbon hydroxylase in human lymphocytes. *Am. J. Hum. Genet.*, **25**, 327.

KOREMAN, S.G. & DUKES, B.A. (1970). Specific estrogen binding by the cytoplasm of human breast carcinoma. *J. Clin. Endocrinol. Metab.*, **30**, 639.

KOURI, R.E., MCKINNEY, C.E., SLOMIANY, D.J., SNODGRASS, D.R., WRAY, N.P. & MCLEMORE, T.L. (1982). Positive correlations between high aryl hydrocarbon hydroxylase activity and primary lung cancer as analyzed in cryopreserved lymphocytes. *Cancer Res.*, **42**, 5030.

LEVINE, A.S., MCKINNEY, C.E., ECHELBERGER, C.K., KOURI, R.E., EDWARDS, B.K. & NEBERT, D.W. (1984). Aryl hydrocarbon hydroxylase inducibility among primary relatives of children with leukemia or solid tumors. *Cancer Res.*, **44**, 358.

LYNCH, H.T., ALBANO, W.A., DANES, B.S. & 9 others (1984). Genetic predisposition to breast cancer. *Cancer*, **53**, 612.

MASON, M.E. & OKEY, A.B. (1981). Aryl hydrocarbon hydroxylase activity in mouse, rat, and human mammary tumors. *Cancer Res.*, **41**, 2778.

METTLIN, C. (1984). Diet and the epidemiology of human breast cancer. *Cancer*, **53**, 605.

NEBERT, D.W. & GELBOIN, H.V. (1968). Substrate-inducible microsomal aryl hydroxylase in mammalian cell culture. I. Assay and properties of the induced enzyme. *J. Biol. Chem.*, **243**, 6242.

NEBERT, D.W. & GONZALEZ, F.J. (1987). P450 Genes: Structure, evolution, and regulation. *Ann. Rev. Biochem.*, **56**, 945.

PASANEN, M., STACEY, S., LYKKESFELDT, A., BRIAND, P., HINES, R. & AUTRUP, H. (1988a). Induction of cytochrome P-450IA1 gene expression in human breast tumor cell lines. *Chem-Biol. Interactions*, **66**, 223.

PASANEN, M., STENBÄCK, F., PARK, S.S., GELBOIN, H.V. & PELKONEN, O. (1988b). Immunohistochemical detection of human placental cytochrome P-450-associated monooxygenase system inducible by maternal cigarette smoking. *Placenta*, **9**, 267.

PELKONEN, O. & NEBERT, D.W. (1982). Metabolism of polycyclic aromatic hydrocarbons: etiologic role in carcinogenesis. *Pharmacol. Rev.*, **34**, 189.

PETERSON, G.L. (1977). A simplification of the protein assay method of Lowry et al., which is more generally applicable. *Anal. Biochem.*, **83**, 346.

ROBERTS, E.A., GOLAS, C.L. & OKEY, A.B. (1986). Ah receptor mediating induction of aryl hydrocarbon hydroxylase: detection in human lung by binding of 2,3,7,8-[$^3$H]tetrachlorodibenzo-p-dioxin. *Cancer Res.*, **46**, 3739.

ROBINSON, S.P. & JORDAN, V.C. (1988). Metabolism of steroid-modifying anticancer agents. *Pharmac. Ther.*, **41**, 41.

SABADIE, N., RICHTER-REICHHELM, H.B., SARACCI, R., MOHR, U. & BARTSCH, H. (1981). Inter-individual differences in oxidative benzo(a)pyrene metabolism by normal and tumorous surgical lung specimens from 105 lung cancer patients. *Int. J. Cancer.*, **27**, 417.

SENLER, T.I., DEAN, L.W.L., MURRAY, L.F. & WITTLIFF, J.L. (1985a). Quantification of cytochrome P-450-dependent cyclohexane hydroxylase activity in normal and neoplastic reproductive tissues. *Biochem. J.*, **227**, 387.

SENLER, T.I., HOFMANN, G.E., SANFILIPPO, J.S., BARROWS, G.H., DEAN, W.L., WITTLIFF, J.L. (1985b). Cytochrome P-450 activity in human leiomyoma and normal myometrium. *Am. J. Obstet. Gynecol.*, **153**, 551.

THOMAS, D.B. (1984). Do hormones cause breast cancer? *Cancer*, **53**, 595.

WILKINSON, L. (1986). *Systat: The System for Statistics*. Evanston, IL: Systat, Inc.